# Opening doors to clinical trial participation among Hispanics: Lessons learned from the Spanish translation of ResearchMatch

**DOI:** 10.1017/cts.2020.539

**Published:** 2020-09-11

**Authors:** Loretta M. Byrne, Sarah K. Cook, Nan Kennedy, Michael Russell, Rebecca N. Jerome, Jason Tan, Claudia Barajas, Consuelo H. Wilkins, Paul A. Harris

**Affiliations:** 1 Vanderbilt Institute for Clinical and Translational Research, Vanderbilt University Medical Center, Nashville, TN, USA; 2 Vanderbilt-Ingram Cancer Center, Vanderbilt University Medical Center, Nashville, TN, USA; 3 Department of Medicine, Vanderbilt University Medical Center and Department of Internal Medicine, Meharry Medical College, Nashville, TN, USA; 4 Office of Health Equity, Vanderbilt University Medical Center, Nashville, TN, USA; 5 Department of Biomedical Informatics, Vanderbilt University Medical Center, Nashville, TN, USA

**Keywords:** Spanish translation, health literacy, Hispanic recruitment, advisory panel, participant enrollment, community engagement

## Abstract

**Introduction:**

Clinical trial participation among US Hispanics remains low, despite a significant effort by research institutions nationwide. ResearchMatch, a national online platform, has matched 113,372 individuals interested in participating in research with studies conducted by 8778 researchers. To increase accessibility to Spanish speakers, we translated the ResearchMatch platform into Spanish by implementing tenets of health literacy and respecting linguistic and cultural diversity across the US Hispanic population. We describe this multiphase process, preliminary results, and lessons learned.

**Methods:**

Translation of the ResearchMatch site consisted of several activities including: (1) improving the English language site’s reading level, removing jargon, and using plain language; (2) obtaining a professional Spanish translation of the site and incorporating iterative revisions by a panel of bilingual community members from diverse Hispanic backgrounds; (3) technical development and launch; and (4) initial promotion.

**Results:**

The Spanish language version was launched in August 2018, after 11 months of development. Community input improved the initial translation, and early registration and use by researchers demonstrate the utility of Spanish ResearchMatch in engaging Hispanics. Over 12,500 volunteers in ResearchMatch self-identify as Hispanic (8.5%). From August 2018 to March 2020, 162 volunteers registered through the Spanish language version of ResearchMatch, and over 500 new and existing volunteers have registered a preference to receive messages about studies in Spanish.

**Conclusion:**

By applying the principles of health literacy and cultural competence, we developed a Spanish language translation of ResearchMatch. Our multiphase approach to translation included key principles of community engagement that should prove informative to other multilingual web-based platforms.

## Introduction

While people of Hispanic background comprise approximately 18% of the US population, less than 8% of clinical trial participants identify as Hispanic [1]. The National Institutes of Health (NIH) recognized this shortcoming and mandated that clinical research investigators make a substantial effort to recruit minorities, including this group [2]. Large academic medical centers with NIH-funded Clinical and Translational Science Awards (CTSAs) have been charged with improving Hispanic enrollment in trials [3], and university and hospital ethics boards endorse the importance of boosting minority inclusion [4].

Language barriers represent an issue of particular importance in clinical trial recruitment. The geographic origins of the US Hispanic population are heterogeneous, representing more than 20 different Spanish-speaking countries [5], with the most common backgrounds being Mexican (62.9%), Central American (9.5%), Puerto Rican (9.2%), South American (6.4%), and Cuban (3.8%) [6]. Because 38% of US Hispanics are estimated to speak Spanish primarily or exclusively [7], Spanish translations of research-related materials should be universally rendered in a manner that promotes understanding and inclusivity. Variations in dialect and terms, as well as variability in culture, can present translation challenges and require a thoughtful approach to translating English language recruitment content and messaging into Spanish.

**Fig. 1.**
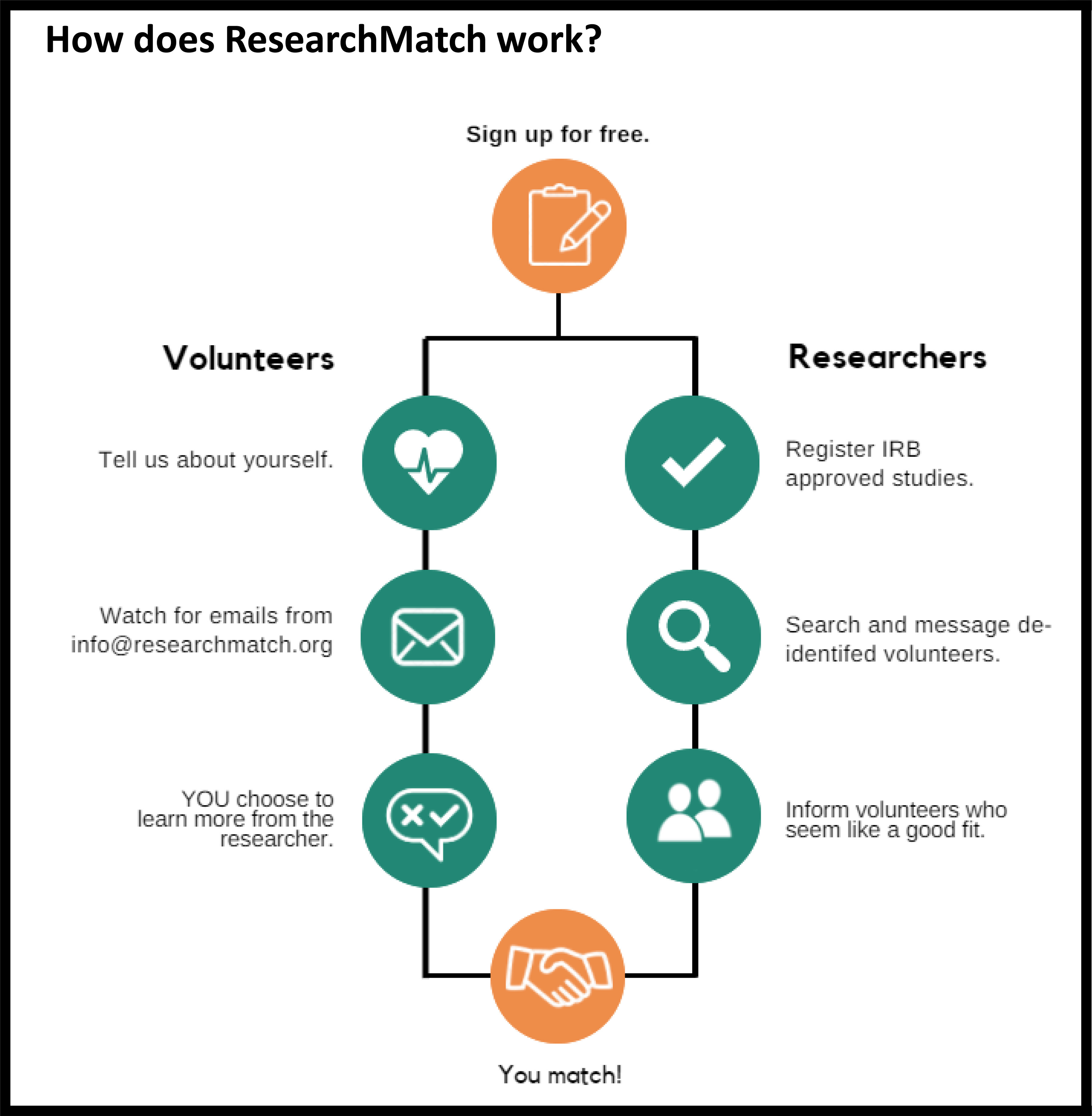
The ResearchMatch approach to matching researchers with volunteers.

ResearchMatch [8] is an online platform that facilitates the awareness of and the participation in research by connecting individuals with researchers seeking participants, via a secure, online interface (Fig. 1). Created in 2009 at Vanderbilt University Medical Center (VUMC) with the collaboration of the CTSA consortium of institutions and funded through the National Center for Advancing Translational Sciences (NCATS), ResearchMatch currently supports over 146,500 self-registered volunteers, and 8000 researchers from 169 research institutions.

Creating a Spanish language version of ResearchMatch would, we hoped, extend this platform’s functionality by increasing awareness of research opportunities and lowering the participation barriers for Spanish-speaking populations by connecting them with researchers. This paper describes the methods we used to create a Spanish translation of ResearchMatch, and documents lessons learned, in hopes of guiding others looking to create content relevant to diverse populations. We also share initial results that demonstrate the early success of the Spanish platform.

## Methods

### Phase 1: Preparing Content

Beginning in 2017, we sought to follow the health literacy and capacity building guidance of the US Department of Health and Human Services (HHS) [9] and the Healthy People 2010 and 2020 framework [10] by examining the existing ResearchMatch online content in the English language before beginning our Spanish translation work. In keeping with HHS’s Office of Minority Health’s, National Standards for Culturally and Linguistically Appropriate Services guidelines [11,12], our subsequent translation methods sought to produce a dialect-neutral translation that was culturally congruent with and respectful to a broad range of Spanish-speaking cultures.

Before translating, we engaged the services of the Vanderbilt Effective Health Communication Core to simplify the English version by removing jargon, shifting to a conversational tone, shortening sentences, and replacing long or complex words with common one- or two-syllable alternatives. By doing so, we achieved a plain language version of the English platform and reduced the literacy level.

Two site components were identified as posing a significant challenge to successful full-site translation. First, *Trials Today at ResearchMatch* [13], is a public-facing clinical trial search engine that automatically extracts information from ClinicalTrials.gov, an English language-only resource. Second, the *Results* page, pulls lists of publications from PubMed.gov where recruitment was supported by ResearchMatch. These references are generally available only in English. We opted to include these two content areas, even though it was not currently scalable to translate them into Spanish, to better convey inclusivity and transparency.

### Phase 2: Spanish Translation

After receiving approval from the VUMC Institutional Review Board (IRB), we contracted with a vetted company for a Latin American Spanish translation of ResearchMatch, (Language Lines Solutions, Monterey, CA). English text provided to the translation company included: web application pages, documents (*Volunteer Agreement*, *Privacy Statement*, *and Terms of Use*), and system-generated email messages (sign-up confirmation and study opportunity contact messages). Services also included copyediting and final proofreading by two or more translators.

### Spanish Translation Advisory Panel

The first body of professionally translated content was reviewed by a Spanish-speaking colleague and the Vanderbilt Recruitment Innovation Center (RIC) Community Advisory Board (CAB). Both suggested that we assemble a diverse Spanish-speaking advisory committee to ensure that our translation reflected the needs, priorities, and values of the broad Spanish-speaking community.

Based on this feedback, we established a Spanish Translation Advisory Panel (STAP) to provide cultural context and relevance to the professional translation, and to ensure the translation was dialect-neutral, inoffensive, and understandable by Spanish speakers from a range of backgrounds, with different dialects and literacy levels. Members of our team had successfully used this approach in the past when developing survey materials for the *All of Us* program [14].

Four native Spanish speakers (1 male, 3 females) ranging in age from early 20s to middle 60s accepted the invitation to take part as STAP members. Each was from a different country of origin (Mexico, Puerto Rico, Guatemala, and Ecuador). All were fluent in both written and spoken English and Spanish, and none had prior exposure to ResearchMatch. One had a background in translation work. STAP members were compensated on an hourly basis as external consultants for their time and transportation costs.

### Advisory Panel Process

The STAP met in person at VUMC or via web conference from December 2017 through June 2018. A ResearchMatch project coordinator, with assistance from a bilingual colleague familiar with ResearchMatch, facilitated the discussions in Spanish and confirmed all STAP edits and recommendations. The coordinator guided the STAP through Spanish language web content to provide context. The panel provided recommendations to improve the clarity and accuracy of the professionally translated text. These discussions were free-flowing, and opinions were sought to describe what the translated text meant to each STAP member to ensure accuracy and consistency in meaning. Once meaning was determined, the members expressed opinions about the reading level, understandability, voice, and any inappropriateness in their native Spanish dialect. When discrepancies of opinion were noted, each member was asked to suggest alternatives. After reaching consensus, back-translations (Spanish to English and comparison with the original version) were performed to ensure the original meaning was maintained. After reviewing the public pages and the volunteer registration workflow, the STAP reviewed the *Volunteer Agreement* and *Privacy Policy*, promotional materials, and a quality improvement survey.

The STAP’s final recommendations and edits were implemented by the ResearchMatch technical developers on a nonpublic prototype web application, thus allowing a final review within the context of the overall website prior to full implementation.

### Phase 3: Technical Development and Launch

#### User interface and application changes

Moving from a single language to a multilingual web application required technical architecture modifications. ResearchMatch is built using the Symfony framework (Symfony SAS, Clichy, France) and a MariaDB (MariaDB Foundation, Delaware, USA) back-end database [8]. Following standard translation methodology, our technical team extracted and replaced all web content callouts with references to single variable text strings (e.g., intro_sentence_en = “Researchers need your help!”; intro_sentence_es = “¡Los investigadores necesitan su ayuda!”). Once abstracted, this content could be used to create files to reference when displaying translated material at the point of the web application load so that ResearchMatch web content was displayed in a selected language (“en = English”; “es = Spanish”) for the end user. Programmatically, text string abstraction allowed our translators to work independently from our developers. While our current initiative was designed around translating ResearchMatch into Spanish, this language abstraction methodology work will also create a straightforward path for rendering ResearchMatch in other languages in the future.

Functionality allowing volunteers to specify their language preference was also added. As part of the sign-up process, volunteers select the language(s) in which they wish to receive email messages from ResearchMatch – in English, Spanish, or both. The ResearchMatch researcher workflow enables rapid cohort selection and messaging to potential volunteers. With the multilingual translation work, we enhanced this communication workflow to enable researchers to supply IRB-approved study recruitment language in either Spanish or English.

#### Implementing UMLS terms for volunteer-reported health conditions and medications

A key feature of the ResearchMatch volunteer onboarding process is asking laypersons to self-report historical medical conditions in a way that can be translated for use by researchers to inform volunteer cohort selection for specific studies (e.g., inclusion/exclusion criteria). To achieve this goal, we leverage the Unified Medical Language System (UMLS), a curated database maintained by the National Library of Medicine that serves to provide interconnectivity and cross-ontology mapping of biomedical terms [15]. For example, laypersons might be more familiar with the term “heart attack” when reporting their health history, while research teams might be more familiar with the term “myocardial infarction” or even “MI”. The UMLS database allows mapping of all three “text strings” as a single computer-friendly concept unique identifier (UMLS CUI = “C0027051”). For the new Spanish translation work in ResearchMatch, we chose to use “text strings” from the Spanish language version of the Systematized Nomenclature of Medicine (SNOMED) [16,17] and MEDLINE [18] as information sources from within the UMLS, enabling potential volunteers to specify health conditions in their preferred language.

Extensive testing was conducted in July to ensure all aspects were complete and functional, and after the site was launched on August 2, 2018, early adopter volunteers who signed up in Spanish were sent a quality improvement survey, assessing the ease of registration and understandability of the translated content (Supplementary Table 1).

### Phase 4: Initial Promotion

Raising local awareness in Nashville, TN included discussions with community health educators, researchers recruiting Spanish speakers, and members of local nonprofit organizations. Early national outreach to promote the use of the Spanish language website included disseminating ResearchMatch Spanish promotional materials, posting on social media, and strategic stakeholder engagement. The latter included meeting with leadership of the NIH Spanish website *Salud NIH*, and informing the ResearchMatch researchers, and the institutional Liaisons who promote the platform and support researchers at each institution. We also asked that the ResearchMatch institutions publicize the new Spanish resource via their websites, which historically have been a significant source of digital referrals to www.ResearchMatch.org.

## Results

### Outcomes of Spanish Translation Process

Much of the professionally translated content was found to require at least one, if not several, iterative modifications by the STAP over the 17 h of meetings. On the home page, 76 of the 247 words (31%) of the initial translation were changed. Some of the more difficult-to-translate text on ResearchMatch underwent a process of “transcreation,” in which content was recreated in Spanish while preserving the meaning, style, tone, and intent of the English text. The panel emphasized that some terms and phrases were not translatable, such as colloquialisms, idioms, and slogans. Examples of these and other revisions made by the STAP are shown in Supplementary Table 2.

The Spanish ResearchMatch site was launched in August 2018. Apart from the aforementioned *Trials Today* and *Results* pages, the translated content provides a user experience that is nearly identical to that of the English site.

With minimal promotion, as of March 3, 2020, 162 people (62% female, 38% male) registered as volunteers through the Spanish ResearchMatch site, with the majority identifying as Hispanic ethnicity (98%) and non-White (62%). Of these, 122 volunteers (75%) have been contacted with information about at least one research study opportunity, with 48 (39%) expressing interest in the study and sharing their contact information with the researcher. Seven research studies have sent at least one Spanish recruitment message to volunteers.

Since the option became available to receive recruitment messages in Spanish, 503 ResearchMatch volunteers have elected to receive contact messages in Spanish (97% were new volunteers; 3% were previously registered volunteers), with 359 (71.4%) electing to receive both Spanish and English contact messages. These numbers represent volunteers who have registered on both the English and Spanish platforms, including those who have updated their language preference to receive contact messages in either “Spanish” or “English and Spanish”.

Differences in the reporting of health conditions were observed among newly registered volunteers. Of the volunteers who registered in Spanish, 64% did not report any health conditions, while 36% reported at least one. In comparison, 33% of volunteers who registered in English during the same period reported no health conditions while 65% reported at least one.

**Fig. 2.**
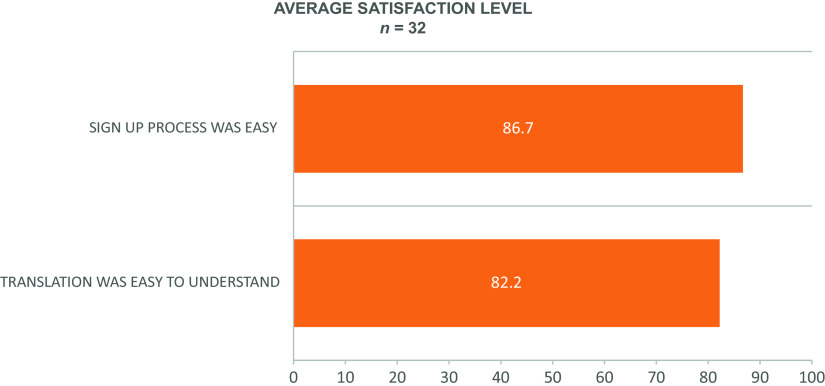
Results from the Spanish ResearchMatch quality improvement survey. Scales of 1–100, where 100 = “totally agree process was easy” and “very easy to understand”.

Thirty-two of the initial volunteers who registered using the Spanish-translated site participated in the quality improvement survey described earlier (Fig. 2).

## Discussion

Although Hispanics have a history of participation in clinical trials [19,20], research shows they are less aware of available trials than non-Hispanics, less likely to understand what a clinical trial is, and less likely to learn about or be invited to participate in clinical trials [21–24]. Despite their potential interest, recruitment of Hispanics remains challenging, with language constituting a major barrier [25,26].

The translation of online clinical trial recruitment registries into Spanish has been undertaken by others with mixed success [27,28]. A ClinicalTrials.gov Spanish language prototype was attempted over a decade ago [29], but was not moved into full production. The site managers encountered insurmountable challenges, including the need for Spanish-speaking support staff, more technical resources, and ongoing translation, extraction, and synchronization of information [29].

Various papers have reported on the use of community engagement and review panels to achieve culturally-sensitive Spanish translations of health-related materials [30–34]. The method we used was similar to a patient-centered website that was translated into Spanish to educate Hispanics on living donor kidney transplantation. Researchers in that case used focus groups with Hispanic patients, family, stakeholders, and the general public to incorporate feedback on patient needs and cultural values [35].

Our method differed, however, in that we relied on the Spanish Language Advisory Panel for frequent and consistent translation insights throughout the process. We not only employed a multi-prong approach to include many best practices for translation [30–33], including forward and backward translations, review by bilingual health professionals, transcreation, pilot testing, and simplifying source material, but we sought out each STAP member’s individual opinions and cultural preferences, arriving at final decisions only through consensus. This thorough approach demonstrates respect for the individual Spanish translator, recognizes the individual’s importance in developing and informing this resource, and underscores the value of the community’s participation in research.

Regarding the early response to the new platform, the Spanish-speaking registrants who completed the satisfaction survey reported the process of joining as easy and the content easy to understand. Reaction from our institutional Liaisons has been enthusiastic, with stakeholders expressing appreciation for the translated site as a means for connecting monolingual Spanish speakers with research opportunities in a way that did not previously exist.

Interestingly, the reporting of health conditions by the volunteers joining via the Spanish-translated ResearchMatch site was much lower than that of new volunteers registering using the English ResearchMatch. Volunteers are not required to report conditions and may not have health conditions to report. However, this cohort may have been reticent or mistrustful, the translation may have inadvertently affected this outcome, or the health condition page may have posed other difficulties for them. We will monitor to see if this trend continues, and potentially engage in gathering additional user feedback if the signal persists.

### Limitations

There are several limitations to note. The STAP was representative of only four of the five major regional backgrounds of origin comprising the US Hispanic population: Mexican, Central American, Puerto Rican, and South American. We were unable to secure a panel member from the Cuban background and thus, the translation is lacking this influence. The reading level of the web application remains above a 6^th^-grade reading level due to some content complexity.

ResearchMatch volunteers require email access and the ability to self-register or be registered by a parent or guardian. Individuals with limited internet and/or email access could be unable to register and respond to contact messages about research studies. However, aspects of the digital divide among ethnic groups have been narrowing. For example, although fewer Hispanics than Whites report owning a personal computer (57% vs. 82%), equal numbers of the 2 groups (about 8 in 10) own a smartphone. Even among US Hispanics born outside the country, smartphone ownership approaches 70% [36].

The matching of volunteers to researchers is contingent on many factors, most importantly, the nature of the study and the content of the recruitment message. Given the complexity of a quality Spanish translation, we may see poorly translated content in the Spanish messages sent by the researchers.

### Lessons Learned

We found that the use of a professional translation company was necessary but insufficient and that the process of translating into Spanish was more time-intensive than initially anticipated, largely due to the amount of content that our assembled STAP needed to review and rework.

Engagement of a diverse, multicultural panel of fluent Spanish speakers ensured the translated web application was dialect-neutral, grammatically correct, and “made sense” – being congruent with the meaning and tone of the English site, while also being consistent with Hispanic values. By meeting as a group in a discussion-based format, the panel was able to offer invaluable insight that was not necessarily feasible or accessible through a standard translation company service.

Moreover, to ensure the lasting engagement of the STAP members, it was important to schedule convenient and accessible meetings, provide adequate time to review materials, and provide fair, competitive, and timely compensation. The inclusion of at least one bilingual staff member to assist with the facilitation of STAP meetings was crucial. Staff investment included considerable hours for planning, implementation, and project management.

The information technology requirements to develop the new site and its interface with the original English site were extensive and time-intensive. To mitigate technical issues, we engaged in ongoing and proactive testing. Technical staff investment included abstracting language and building a new multilingual workflow.

Most importantly, we recognize that registration at the time of writing is low, and that increasing the percentage of Spanish-speaking volunteers in ResearchMatch is a challenge which requires more than merely translating the web application. Strategic efforts to raise awareness are planned and will need to involve allied community and health professionals and national partners. However, there are larger issues that are beyond just awareness-raising of this platform. Additional barriers must be addressed for study participation to increase among Hispanics, including limited awareness of clinical trials, strict inclusion or exclusion criteria, lack of availability due to conflicting demands, fear of harm, lack of insurance coverage to pay for treatment, mistrust, and fears regarding residency status. Some of these factors may contribute to lower ResearchMatch registration rates as well.

#### Future Directions – increasing participation in biomedical research by Spanish speakers

While these new volunteers represent a small subset of the overall ResearchMatch volunteer community, their action in joining the site suggests that a Spanish version of ResearchMatch is valued and appreciated. Many factors will affect not only the potential number of new registrations but also the use of the translated platform by researchers. Although many of these barriers may be beyond the scope of this project, efforts are being made to raise awareness of the site itself.

To expand awareness, we will foster relationships with nonprofit patient advocacy partners and organizations whose purpose is to provide agency and resources to the Hispanic community. Sustainability will include a commitment to incorporate Spanish content in all communications and future updates to ResearchMatch. Customer service assistance is now available in Spanish and we will create Spanish language videos demonstrating the sign-up process.

Web application performance and effectiveness will be assessed by monitoring usage metrics and making necessary modifications. For example, it may be useful to compare response rates to recruitment messages among English versus Spanish-speaking volunteers and to examine how registrations and drop-off rates for the Spanish portal compare to the English language portal. To plan more targeted approaches to engage Spanish-speaking individuals to join the ResearchMatch community, a deeper assessment of the geographic areas where Spanish-speaking volunteers are concentrated may be needed to determine whether ample of clinical trial opportunities are readily available.

## Conclusion

Through a combination of partnering with Spanish-speaking community members as project consultants, applying the principles of health literacy and cultural and linguistic competency, rigorous vetting and matching of medical terminology, and state-of-the-art information technology approaches, we developed a Spanish language version of ResearchMatch. Our STAP of native Spanish speakers was selected from a range of Hispanic backgrounds, and their approach of iteratively refining our translation was innovative. We learned that producing a successful translation of a health-related web application from English to Spanish requires substantial investment in time and resources if the translated site is to be functional and easily understood by a heterogeneous population of Spanish speakers, while at the same time culturally sensitive. We believe our detailed approach and lessons learned will be instructive to others who wish to engage in similar translation efforts. Preliminary data on usage of the translated site and increased site enrollment by Hispanics are encouraging and may open the door to greater clinical trial participation among Spanish speakers.

## Supplementary material

For supplementary material accompanying this paper visit https://doi.org/10.1017/cts.2020.539. click here to view supplementary material

## References

[1] Global Institute for Hispanic Health working to increase clinical trial participation. Driscoll Children’s Hospital [Internet] [cited 2019 Jul 9]. (http://www.driscollchildrens.org/about-us/global-institute-for-hispanic-health-working-to-increase-clinical-trial-participation)

[2] NIH Policy and Guidelines on The Inclusion of Women and Minorities as Subjects in Clinical Research [Internet] [cited 2018 Oct 30]. (https://grants.nih.gov/grants/funding/women_min/guidelines.htm)

[3] CTSA-Program-Collaborative-Innovation-Suite-of-Awards-Resource-Booklet-10.2017.pdf [Internet] [cited 2018 Oct 30]. (https://ctsa.ncats.nih.gov/wp-content/uploads/2018/01/CTSA-Program-Collaborative-Innovation-Suite-of-Awards-Resource-Booklet-10.2017.pdf)

[4] Taylor HA . Inclusion of women, minorities, and children in clinical trials: opinions of research ethics board administrators. Journal of Empirical Research on Human Research Ethics (JERHRE) 2009; 4(2): 65–73. 19480593 10.1525/jer.2009.4.2.65 PMC2859460

[5] Lopez MH , Gonzalez-Barrera A , Cuddington D . Diverse Origins: The Nation’s 14 Largest Hispanic-Origin Groups. Pew Research Center [Internet], 2013 [cited 2018 Nov 16]. (http://www.pewhispanic.org/2013/06/19/diverse-origins-the-nations-14-largest-hispanic-origin-groups/)

[6] Bureau UC . The Hispanic Population in the United States: 2016 [Internet] [cited 2018 Oct 30]. (https://www.census.gov/data/tables/2016/demo/hispanic-origin/2016-cps.html)

[7] Krogstad JM , Gonzalez-Barrera A . A majority of English-speaking Hispanics in the U.S. are bilingual [Internet]. Pew Research Center [cited 2020 Aug 3]. (https://www.pewresearch.org/fact-tank/2015/03/24/a-majority-of-english-speaking-hispanics-in-the-u-s-are-bilingual/)

[8] Harris PA , et al. Research match: a national registry to recruit volunteers for clinical research. Acad Med J Assoc Am Med Coll. 2012; 87(1): 66–73. 10.1097/ACM.0b013e31823ab7d2 PMC3688834 22104055

[9] National Action Plan to Improve Health Literacy | health.gov [Internet], 2010 [cited 2020 Feb 10]. (https://health.gov/our-work/health-literacy/national-action-plan-improve-health-literacy)

[10] Healthy People 2020 Framework. US Preventive Services Task Force, United States. Office of Disease Prevention, and Health Promotion. 2020; 3.

[11] Culturally and Linguistically Appropriate Services [Internet]. Think Cultural Health [cited 2020 Feb 10]. (https://thinkculturalhealth.hhs.gov/)

[12] Andrulis DP , Brach C . Integrating literacy, culture, and language to improve health care quality for diverse populations. American Journal of Health Behavior 2007; 31(Suppl 1): S122–S133. 17931131 10.5555/ajhb.2007.31.supp.S122 PMC5091931

[13] Jerome RN , et al. To end disease tomorrow, begin with trials today: digital strategies for increased awareness of a clinical trials finder. Journal of Clinical and Translational Science 2019; 3(4): 190–198. 31660243 10.1017/cts.2019.404 PMC6799228

[14] Cronin RM , et al. Development of the initial surveys for the all of us research program. Epidemiology 2019; 30(4): 597–608. 31045611 10.1097/EDE.0000000000001028 PMC6548672

[15] Unified Medical Language System (UMLS) [Internet] [cited 2019 Jun 10]. (https://www.nlm.nih.gov/research/umls/)

[16] Pérez M , Berlanga R . Semantic transference for enriching multilingual biomedical knowledge resources. Journal of Biomedical Informatics 2015; 58: 1–10. 26386313 10.1016/j.jbi.2015.08.026

[17] Hellrich J , Hahn U . Fostering multilinguality in the UMLS: a computational approach to terminology expansion for multiple languages. AMIA Annual Symposium Proceedings 2014; 2014: 655–660. 25954371 PMC4419887

[18] MedlinePlus - Información de Salud de la Biblioteca Nacional de Medicina [Internet] [cited 2018 Nov 13]. (https://medlineplus.gov/spanish/)

[19] Garza MA , et al. The influence of race and ethnicity on becoming a human subject: Factors associated with participation in research. Contemporary Clinical Trials Communications 2017; 7: 57–63. 29226266 10.1016/j.conctc.2017.05.009 PMC5716487

[20] Wendler D , et al. Are racial and ethnic minorities less willing to participate in health research? PLoS Medicine 2006; 3(2). 10.1371/journal.pmed.0030019 PMC1298944 16318411

[21] Wallington SF , et al. Assessing the awareness of and willingness to participate in cancer clinical trials among immigrant Latinos. Journal of Community Health 2012; 37(2): 335–343. 21805372 10.1007/s10900-011-9450-y PMC3567194

[22] Arevalo M , et al. Mexican-American perspectives on participation in clinical trials: a qualitative study. Contemporary Clinical Trials Communications 2016; 4: 52–57. 27570845 10.1016/j.conctc.2016.06.009 PMC4999069

[23] Ford ME , et al. Unequal burden of disease, unequal participation in clinical trials: solutions from African American and Latino Community Members. Health & Social Work 2013; 38(1): 29–38. 23539894 10.1093/hsw/hlt001 PMC3943359

[24] Rogers W , Lange MM . Rethinking the vulnerability of minority populations in research. American Journal of Public Health 2013; 103(12): 2141–2146. 24134375 10.2105/AJPH.2012.301200 PMC3828952

[25] Ulrich A , et al. Issues in biomedical research: what do Hispanics think? American Journal of Health Behavior 2013; 37(1): 80–85. 22943104 10.5993/AJHB.37.1.9 PMC3860276

[26] George S , Duran N , Norris K . A systematic review of barriers and facilitators to minority research participation among African Americans, Latinos, Asian Americans, and Pacific Islanders. American Journal of Public Health 2014; 104(2): e16–e31. 10.2105/AJPH.2013.301706 PMC3935672 24328648

[27] DS-Connect Registry [Internet] [cited 2018 Nov 16]. (https://dsconnect.nih.gov/)

[28] The Cerebral Palsy Research Registry en Español [Internet] [cited 2018 Nov 16]. (https://www.cpregistry.org/espanol.php)

[29] Rosemblat G , Tse T . User study of a Spanish-language ClinicalTrials.gov prototype system. AMIA Annual Symposium Proceedings 2006; 2006: 659–663. PMC1839723 17238423

[30] Solomon FM , et al. Development of a linguistically and culturally appropriate booklet for Latino cancer survivors: lessons learned. Health Promotion Practice 2005; 6(4): 405–413. 16210682 10.1177/1524839905278447

[31] Forcino RC , et al. Developing and pilot testing a Spanish translation of CollaboRATE for use in the United States. PLoS One 2016; 11(12). 10.1371/journal.pone.0168538 PMC5176178 28002422

[32] Butler SF , et al. Spanish translation and linguistic validation of the Screener and Opioid Assessment for Patients with Pain-Revised (SOAPP-R). Pain Medicine 2013; 14(7): 1032–1038. 23590454 10.1111/pme.12098

[33] Brelsford KM , Ruiz E , Beskow L . Developing informed consent materials for non-English-speaking participants: an analysis of four professional firm translations from English to Spanish. Clinical Trials 2018; 15(6): 557–566. 30295050 10.1177/1740774518801591 PMC6218315

[34] Rhodes SD , et al. Selling the product: strategies to increase recruitment and retention of Spanish-speaking Latinos in biomedical research. Journal of Clinical and Translational Science 2018; 2(3): 147–155. 30510779 10.1017/cts.2018.314 PMC6269095

[35] Gordon EJ , et al. A culturally targeted website for Hispanics/Latinos about living kidney donation and transplantation: a randomized controlled trial of increased knowledge. Transplantation 2016; 100(5): 1149–1160. 26444846 10.1097/TP.0000000000000932

[36] Perrin A , Turner E . Smartphones help blacks, Hispanics bridge some – but not all – digital gaps with whites [Internet]. Pew Research Center, 2019 [cited 2020 Feb 10]. (https://www.pewresearch.org/fact-tank/2019/08/20/smartphones-help-blacks-hispanics-bridge-some-but-not-all-digital-gaps-with-whites/)

