# Supplementary material for: Opening doors to clinical trial participation among Hispanics: Lessons learned from the Spanish translation of ResearchMatch
Source: J Clin Transl Sci. 2020 Sep 11;5(1):e46. doi: 10.1017/cts.2020.539 (PMC8057389; doi:10.1017/cts.2020.539)
Supplement: Supplementary file 1 [file S2059866120005397sup.zip › S2059866120005397sup001.docx]

**Supplementary Table 2. Examples of website content, first professional translation, and final revisions by the SPAP**

| **English website verbiage** | **First translation** | **Revised translation by**  **Advisory Panel** | **Context** |
| --- | --- | --- | --- |
| How to get started | Cómo comenzar | Cómo empezar | ‘Comenzar’ and ‘empezar’ both mean ‘to start.’ In this case, ‘empezar’ was deemed more appropriate. |
| Health research changes people’s lives every day | La investigacion sobre la salud cambia las vidas de las personas a diario | La investigación sobre la salud cambia la vida de las personas a diario. | The more literal translation read as the ‘lives of people’ implying that a person has multiple lives. The APAP changed ‘lives’ to ‘life.’ |
| Difficult diseases have met their match | Enfermedades difíciles han encontrados su competencia | None | ‘Met their match’ is an untranslatable English idiom. We decided to remove this sentence from both the English and Spanish sites. |
| Help us spread the word about ResearchMatch.org by sharing with your family and friends | Ayúdenos a correr la voz sobre ResearchMatch.org al compartir con su amigos y familia | Ayúdenos a correr la voz sobre ResearchMatch.org al compartir con su familia y amigos | The panel advised us that family relationships in Hispanic cultures are more important than friends, so the order was reversed. |
