# Supplementary material for: Opening doors to clinical trial participation among Hispanics: Lessons learned from the Spanish translation of ResearchMatch
Source: J Clin Transl Sci. 2020 Sep 11;5(1):e46. doi: 10.1017/cts.2020.539 (PMC8057389; doi:10.1017/cts.2020.539)
Supplement: Supplementary file 1 [file S2059866120005397sup.zip › S2059866120005397sup002.docx]

**Supplementary Table 1. Spanish ResearchMatch Quality Improvement Survey (English version)**

**Instructions:** We are looking for feedback about the ResearchMatch signup process. The information you give us will help ResearchMatch improve and make the website easier to use for Spanish-speaking volunteers. Your responses will be anonymous. Thank you for your time and feedback!

If you would like to be entered into the drawing for a ResearchMatch t-shirt, we will ask for your email address at the end of the survey.

**Description:** For the following questions, please use the slider to let us know if you strongly disagree or strongly agree with the statements.

1. **The sign-up process was easy.**

Strongly Disagree 🡨------------------------------------------🡪 Strongly Agree

1. **What sections were hard for you to complete? (check all that apply)**
   - Reading Volunteer Agreement
   - Entering demographic (like age and race) and contact information
   - Creating a username and password
   - Entering security questions
   - Entering health conditions
   - Entering medications
   - Other (please describe):
2. **How understandable is the Spanish Translation?**

Very Hard to Understand 🡨---------------------------------🡪 Very Easy to Understand

1. **What sections were hard to understand? (check all that apply)**
   - Reading Volunteer Agreement
   - Entering demographic (like age and race) and contact information
   - Creating a username and password
   - Entering security questions
   - Entering health conditions
   - Entering medications
   - Other (please describe):
2. **Were you able to enter in your health conditions?**
   - Yes
   - No
   - I don't have any health conditions
   1. **Please explain any issues you came across while trying to enter your health conditions:**
3. **I joined ResearchMatch because: (Please check all that apply)**

- I am looking to get involved in a specific type of research study.
- I am interested in getting paid to participate in a research study.
- I saw information about ResearchMatch on another organization’s website.
- I want to learn more about the types of research studies available to me.
- A friend or family member recommended I join.
- I saw it on Facebook and/or Twitter.
- I wanted to help.
- Other (please describe):

If you would like to tell us more about your experience or help spread the word about ResearchMatch, please email us at [info@researchmatch.org](mailto:info@researchmatch.org)
